# *Anaplasmataceae *and *Borrelia burgdorferi sensu lato *in the sand lizard *Lacerta agilis *and co-infection of these bacteria in hosted *Ixodes ricinus *ticks

**DOI:** 10.1186/1756-3305-4-182

**Published:** 2011-09-20

**Authors:** Anna Ekner, Krzysztof Dudek, Zofia Sajkowska, Viktória Majláthová, Igor Majláth, Piotr Tryjanowski

**Affiliations:** 1 Department of Behavioural Ecology, Adam Mickiewicz University, Umultowska 89, 61-614 Poznań, Poland; 2 Parasitological Institute, Slovak Academy of Sciences, Hlinkova 3, 040-01 Košice, Slovakia; 3 Institute of Zoology, Poznań University of Life Sciences, Wojska Polskiego 71 c, 60-625 Poznań, Poland

**Keywords:** Tick-borne pathogens, Reptiles, Mixed infection, *Lacertidae*, Co-occurence

## Abstract

**Background:**

*Anaplasmataceae *and *Borrelia burgdorferi *s.l. are important tick-borne bacteria maintained in nature by transmission between ticks and vertebrate hosts. However, the potential role of lizards as hosts has not been sufficiently studied.

**Results:**

The current study showed that 23 of 171 examined sand lizards *Lacerta agilis *were PCR positive for *Anaplasmataceae*. The nucleotide sequences of the several selected PCR products showed 100% homology with *Anaplasma *spp. found in *Ixodes ricinus *collected in Tunisia and Morocco (AY672415 - AY672420). 1.2% of lizard collar scale samples were PCR positive for *B. lusitaniae*. In addition, 12 of 290 examined *I. ricinus *were PCR positive for *B. burgdorferi *s.l. and 82 were PCR positive for *Anaplasmatacea*. The number of ticks per lizard and the number of ticks PCR positive for both microorganisms per lizard were strongly correlated. Moreover, we found a significant correlation between numbers of ticks infected with *Anaplasmataceae *and with *B. burgdorferi *s.l. living on the same lizard. However, there was no significant correlation between detection of both bacteria in the same tick.

**Conclusions:**

To the best of our knowledge, this is the first report of *Anaplasmataceae *DNA and additionally the second report of *B. burgdorferi *s.l DNA detection in the sand lizard.

## Background

One of the most widespread bacterium transmitted by ticks is *Borrelia burgdorferi *s. l., an agent of Lyme borreliosis [1,2]. Reservoirs of *B. burgdorferi *s.l. are vertebrates and special associations between *Borrelia *strains and particular groups of vertebrate hosts have been reported [3]. *B. lusitaniae *was the most common strain detected in lizard species and in ticks feeding on them [4-6].

Ticks are the main vector of other microorganisms, such as intracellular bacteria from the family *Anaplasmataceae *[7,8], which attract the attention of public health professionals worldwide. One of the most important species of this family is *Anaplasma phagocytophilum *which causes human anaplasmosis (HA), formerly known as a human granulocytic ehrlichiosis (HGE) [9,10]. *A. phagocytophilum *is an obligate intracellular bacterium infecting the neutrophils of various mammalian species [9]. *Anaplasmataceae *are maintained in nature by transmission between vectors and reservoirs. Reservoirs of the bacteria are vertebrates, mainly rodents and ruminants [11,12]. The potential role of reptiles as hosts or reservoirs is not known and has not been sufficiently evaluated. To date, despite being found in ticks feeding on reptiles [13-16], *Anaplasma *spp. has been detected only in three lizard species, *Sceloporus ocidentalis*, *S. graciosus *and *Elgaria coeruleus*, living in North America [13].

The most common species of ticks in Europe, *Ixodes ricinus *[17] feeds on a wide variety of vertebrate hosts, such as mammals and birds [8,18-22]. It also parasitises reptile species, and larvae and nymphs often feed on lizards [5,23-26]. *I. ricinus *may be infected simultaneously with different combinations of bacteria [10,27-29]. Observed microorganism co-infection rates suggest that the risk of infection with one tick-borne bacterium is not independent of other bacteria [30]. There are only a few studies on co-infection of bacteria in wild vector or host populations [31,32]. In addition, as far as we are aware, there has been only one study on co-infection of *Anaplasmataceae *and *B. burgdorferi *s.l. in ticks feeding on lizard species, moreover, on *Lacerta viridis *[16].

In this paper we show the role of sand lizard (*Lacerta agilis*) in the transmission cycle of important tick-borne pathogens, *Anaplasmataceae *and *Borrelia burgdorferi *s.l. Moreover, we found a significant correlation between numbers of ticks infected with *Anaplasmataceae *and with *B. burgdorferi *s.l. living on the same lizard.

## Results

**Figure 1:**
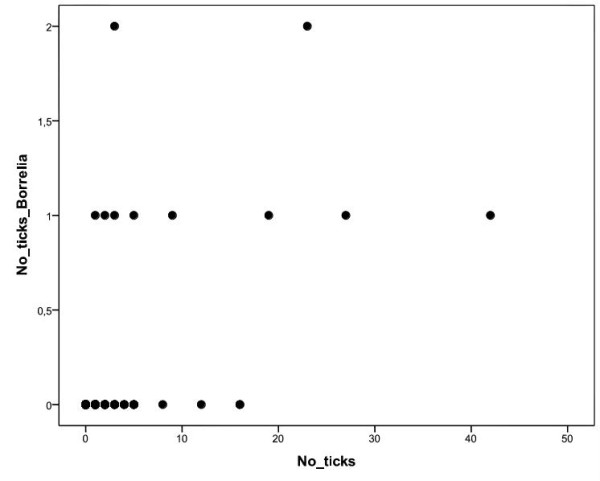
**Correlation between total number of ticks and the ticks PCR positive for *B. burgdorferi *s.l.**. Positive correlation between the number of the ticks feeding on a lizard and the number of the ticks PCR positive for *B. burgdorferi *s.l. (r = 0.57, n = 171, p < 0.0001).

290 ticks (176 nymphs and 114 larvae) were found in 69 of 171 (40.4%, 95% CL: 32.9 - 48.1) examined lizards. All of them were identified as *I. ricinus*. *B. burgdorferi *s.l. DNA was detected in 12 of 290 ticks (4.1%, 95% CL: 2.2 - 7.1; Table 1) taken from 10 lizards (5.9%, 95% CL: 2.8 - 10.5; Table 2). *B. lusitaniae *DNA was detected in 8 ticks (66.7%, 95% CL: 34.9 - 90.1), *B. burgdorferi *s.s. DNA in 2 ticks (16.7%, 95% CL: 02.1 - 48.4), the remaining two strains were not determined in RFLP analyses (Table 1). Among 171 examined lizards (43 adult females, 59 adult males, 26 sub-adults, 43 juveniles), 2 individuals (1 female, 1 sub-adult; 1.2%, 95% CL: 0.1 - 4.1) were PCR positive for *B. lusitaniae*. Both of them had ticks at the time of collection (Table 3). No other *Borrelia *strains DNA were detected in lizard collar scales. The number of ticks per lizard was strongly positively correlated with the number of ticks PCR positive for *B. burgdorferi *s.l. (r = 0.57, n = 171, p < 0.0001; Figure 1).

**Table 1:** Number of ticks PCR positive for *B. burgdorferi *s.l. and *Anaplasmataceae*.

	ticks collected from the lizards 290		
	**ticks infected with *Borrelia*** **12**		**ticks infected with *Anaplasmataceae*** **82**

ticks infected with *B. lusitanieae* 8	ticks infected with *B. burgdorferi *s.s. 2	ticks infected with *B. burgdorferi *s.l. 4	

**Table 2:** Number of lizards with and without ticks PCR positive for *Borrelia *and *Anaplasmataceae*.

lizards without ticks 102		lizards with ticks 69	
	lizards infected with *Anaplasmataceae* 29	lizards infected with *Borrelia* 10	lizards non-infected 30

**Table 3:** Number of lizards PCR positive for *B. burgdorferi *s.l. and *Anaplasmataceae *and presence of ticks in lizards with the bacteria during the time of collection.

lizards cached during the study 171			
**lizards infected with *Borrelia*** **2**		**lizards infected with *Anaplasmataceae*** **23**	

lizards with ticks 2	lizards without ticks 0	lizards with ticks 8	lizards without ticks 15

**Figure 2:**
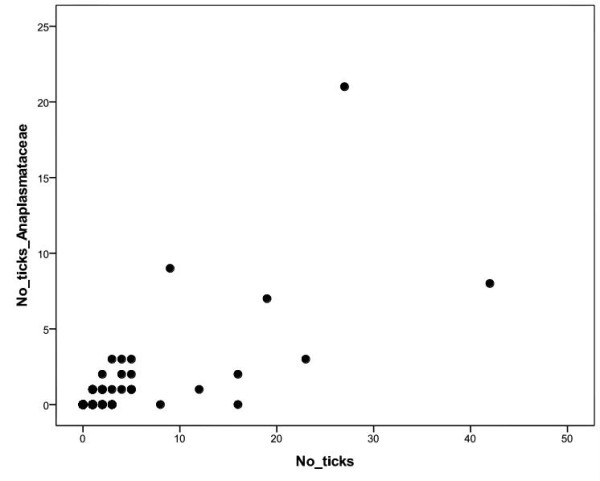
**Correlation between total number of ticks and the ticks PCR positive for *Anaplasmataceae***. Positive correlation between the number of the ticks feeding on a lizard and the number of the ticks PCR positive for *Anaplasmataceae *(r = 0.73, n = 171, p < 0.001).

*Anaplasmataceae *DNA was detected in 82 (52 nymphs, 30 larvae) of 290 ticks (28.3%, 95% CL: 23.2 - 33.8; Table 1) taken from 29 lizards (17.0%, 95% CL: 11.7 - 23.4; Table 2). In the body scales of 171 examined lizards *Anaplasmataceae *DNA was detected in 23 individuals (13.4%, 95% CL = 8.7 - 19.5). Obtained sequences of several PCR products showed 100% homology to each other. The sequence was compared with GenBank entries by Blast N2.2.13 and revealed 100% homology with *Anaplasma *spp. strains found in *I. ricinus *ticks collected on vegetation in Tunisia and Morocco (AY672415 - AY672420). Among the 23 lizards (14 males, 8 females, 1 sub-adult) PCR positive for *Anaplasmataceae *in the skin, 15 had no ticks (65.2%, 95% CL: 42.7 - 83.6) at the time of collection (Table 3). However, lizards that were PCR positive for *Anaplasmataceae *had a higher average number of ticks (mean ± SE; 5.0 ± 2.2) compared to lizards PCR negative for *Anaplasmataceae *(1.2 ± 0.2; U-test, Z = -2.51, p = 0.012), as well as a higher number of ticks PCR positive for *Anaplasmataceae *(2.2 ± 1.0 *vs*. 0.2 ± 0.1; U-test, Z = -2.68, p = 0.007). The number of ticks per lizard and the number of ticks PCR positive for *Anaplasmataceae *per lizard were strongly correlated (r = 0.73, n = 171, p < 0.001; Figure 2).

Kendall's tau coefficient shows that the number of ticks PCR positive for *Anaplasmataceae *were strongly correlated with the number of ticks PCR positive for *B. burgdorferi *s.l. (r = 0.42, n = 171, p < 0.0001) feeding on the same lizard. 6 (2.1%, 95% CL = 0.8 - 04.5) ticks contained both *B. burgdorferi *s.l. and *Anaplasmataceae *DNA. However, Kendall's tau coefficient showed no significant correlation between detection of *B. burgdorferi *s.l. and *Anaplasmataceae *DNA (r = 0.10, n = 290, p = 0.088) in the same tick.

The co-infection index (Ic) for interactions between *B. burgdorferi *s.l. and *Anaplasmataceae *was 0.75. However, the difference between the number of obtained and expected co-infections was not significant (χ^2 ^= 0.02, p = 0.89). This suggested that there was no significant association between the bacteria.

We could not analyse a co-infection in lizards, because of too the small sample of lizards PCR positive for *Borrelia burgdorferi *s.l.

## Discussion

Lizards as hosts of ticks are exposed to various tick-borne pathogens. Previous studies have showed that reptiles are included in transmission cycles of *B. burgdorferi *s. l. [4,5,33]. In our study, *B. lusitaniae *DNA was detected in 1.2% of lizards. Compared with other papers, this is a low infestation [4,5]. However, this is only the second detection of the bacterium in sand lizards [4] and the third detection of the *B. lusitaniae *strain in Poland [6,34]. In all lizards, as well in most of the ticks, *B. lusitaniae *was detected, which confirms the connection of this strain with reptile species [3,35,36].

During the study 28.3% of ticks feeding on lizards were infected with *Anaplasmataceae*. This is high compared to other studies on ticks from lizard species [13,14,16]. However, despite *Anaplasma *spp. DNA being previously detected in ticks collected from reptiles [14], it had only been detected in three lizard species, *S. occidentalis*, *S. graciosus *and *Elgaria coeruleus *[13]. In our study, 13.4% of lizards were PCR positive for *Anaplasmataceae*. To the best of our knowledge this is the first detection of the bacterium in sand lizard, moreover, the first among lizards living in Europe, and only the fourth among lizards worldwide. In addition, the previous report concerned 10.2% of lizards living in California which were infected with *A. phagocytophilum *[13]. In presented study, some lizards were PCR positive for different species of *Anaplasmataceae *than in the previous study on reptiles [13], namely *Anaplasma *spp. 100% homology with strains found in *I. ricinus *collected on vegetation in Tunisia and Morocco (AY672415 - AY672420) [37]. The pathogen could be transferred to Poland together with exotic reptiles or other animals [14]. On the other hand, lizards could not be analysed for presence of that species before.

Lizards PCR positive for *Anaplasmataceae *had more ticks than non infected individuals. Moreover, the number of ticks feeding on a lizard was strongly correlated with both the number of ticks PCR positive for *Anaplasmataceae *and PCR positive for *B. burgdorferi *s.l. It may result from that the more the lizard has ticks, the more chance it has ticks with bacteria. Such correlations suggest that the more ticks feed on a lizard, the greater chance of contact with infected ticks, and hence more chance of acquiring tick-borne infection. The results confirmed previous studies [38,39] which show that the probability of host exposure to a tick-borne pathogen is correlated with tick abundance. Therefore, the best way to avoid infection with tick-borne disease is to avoid areas with a high density of its vectors [18].

Ticks can be infected with two or more microorganisms simultaneously [16,38,40,41], but relationships between them in ticks can be varied. Some of them display antagonistic interactions, others positive, and many evidently do not interact [38]. Interpretation of the results can be difficult, because many factors, other than simply interaction between microorganisms, may influence the number of co-infections. In addition, the occurrence of tick-borne pathogens in nature may be influenced by a number of factors, such as microclimate conditions, vegetation, and tick density [42]. In the present study we did not show any correlation between detection of *B. burgdorferi *s.l. and *Anaplasmataceae*, occurring in the same ticks. The results may suggest a lack of interaction between the bacteria. These results are in contrast to those obtained by Václav [16], where *Anaplasmataceae *had a negative influence on *B. lusitaniae*. However, in the same study, *B. lusitaniae *positively influenced *Anaplasmataceae *prevalence, and co-infection of both bacteria in ticks was higher than expected [16]. To the best of our knowledge, the present work is the first study of mixed infection of *B. burgdorferi *s.l. and *Anaplasmataceae *DNA in ticks feeding on *L. agilis *[16]. Knowledge of the multiple infection is very important for public health, especially for a correct diagnosis and prophylaxis of tick-borne diseases, as well as prognosis of mixed infection in humans. Moreover, hosts infected by several different pathogens can have different symptoms of a disease [43]. Knowledge about the local occurrence of pathogens may be useful when disease symptoms of patients bitten in that locality are unclear [10]. It is important to know how the bacteria can coexist in individual ticks as a prerequisite for the occurrence of co-transmission from tick to the host [30].

In our study, 65.2% of 23 lizards PCR positive for *Anaplasmataceae *did not have any ticks at the time of collection, which may suggest that this bacterium is maintained in a lizard body longer than the source of the infection. However, we still do not know if lizards are reservoirs of *Anaplasmataceae *or just have organisms deposited in them by infected ticks. A previous study, where the author experimentally infected lizards with the bacterium, concluded that lizards were not reservoir hosts for *Anaplasma phagocytophilum *[13]. However, it does not mean that lizards cannot be a reservoir of the other species of *Anaplasmataceae*. Moreover, lizards may influence the transmission cycle of bacteria in areas where there are significant hosts for ticks [2].

## Conclusions

To the best of our knowledge, the current study is the first report of *Anaplasmataceae *DNA, and the second report of *B. burgdorferi *s.l DNA detection in a European lizard species, namely the sand lizard *Lacerta agilis*. Obtained results suggest that lizards may be a reservoirs of this pathogen and can influence the transmission cycle of the bacteria in some areas. Moreover, we found a significant correlation between numbers of ticks infected with *Anaplasmataceae *and with *B. burgdorferi *s.l. living on the same lizard. This knowledge may be important in the estimation of the dispersion of the tick-borne pathogen and/or sources of potential human infection.

## Materials and methods

### Study area and the study species

The study was carried out in March - September in 2008 and 2009 in an extensive farmland area in the Barycz valley, in Poland (51°34'N, 17°40'E, elevation 110-170 m). This study area is characterised by intensively farmed land with a varied mosaic of arable fields, meadows, small woodlots and scattered trees and shrubs of different ages, dominated by white willow *Salix fragilis*, silver birch *Betula pendula*, black poplar *Populus nigra *and pine *Pinus silvestris*. It contains both dry sandy areas and moist areas (for details see reference [44]).

The sand lizard is a short-legged, rather robust, small to medium sized lizard (up to 110 mm snout to vent length (SVL)) from the family *Lacertidae*. It is a ground-dwelling and strongly diurnal species with one of the widest distribution ranges of all reptiles [45]. In the study area the sand lizard is a common species, and an average of 0.37 individuals were noted on 200 m transect route [44].

### Lizard and tick sampling

Lizards were captured using landing fishnets or by hand, then aged (adult, sub-adult and juvenile) and sexed. Animals were examined for the presence of ticks, which were removed with forceps and stored in 70% ethanol. Ticks were identified to species and aged using a binocular microscope, according to Siuda [17].

From each individual lizard a skin biopsy was taken from collar scales (3-4 mm in length) with sterile scissors and put in separate vials with 70% ethanol. This method had been previously successfully used to detect tick-borne pathogens in reptiles [4,5]. The collar is an extension of the skin, hence this method is only minimally invasive to the lizard. The sample is also easy to obtain. To avoid resampling the same individual, lizards were permanently marking using Medical Cautery Units (unpublished observations).

Lizard capture was carried out according to Polish law and the ethical commission for the study on animals (LKE 12/2007).

### DNA isolation

Immediately prior to extraction, ticks and tissues were dried for 30 min to evaporate the ethanol. Each sample was cut with a disposable sterile scalpel. Genomic DNA from lizard scales and from ticks was isolated by alkaline hydrolysis, according to previous reference [46], with a 30 min. incubation time. Cut samples were incubated in the presence of 100 μl ammonium hydroxide (0,5 mol/l) at 100°C for 30 minutes in 1.5 ml tube, followed by 10 minutes at 100°C with the tube open. Isolated DNA was stored at -20°C.

### Polymerase Chain Reaction (PCR)

PCR amplification was performed in a total of 25 μl reaction mixture of a MasterTaq DNA polymerase kit (Eppendorf AG, Hamburg, Germany) containing 10.4 μl of deionized water, 5.0 μl of 5 × TaqMaster PCR Enhancer, 2.5 μl of 10 × *Taq *buffer (with 15 mM Mg^2+^), 1.5 μl of a 25 mM solution of Mg(OAc)2, 0.1 μl of *Taq *DNA polymerase (5 U/ml), 0.5 μl of dNTP-mix (10 mM) (Fermentas, Vilnius, Lithuania), 1.25 μl of each primer (10 pmole/μl) (Invitrogen, Paisley, Scotland), and 2.5 μl of DNA template.

In order to verify that DNA had been successfully isolated from each tick, primers for the fragment of the tick's mitochondrial cytochrome *b *gene (620 bp) were used [47]. Verification of successfully isolated DNA from lizard scales was carried out using primers for the fragment of the vertebrate's 12S rDNA [48]. Seven negative samples of ticks and two negative samples of lizards were excluded from further analysis.

Samples with successfully isolated DNA of ticks and lizard were examined for the presence of *Anaplasmataceae *DNA by amplifying a portion of the region of the 16S (*rrs*) rRNA gene of the family *Anaplasmataceae *[37,49]. Samples were also examined for the presence of *B. burgdorferi *s.l. by amplifying a portion of the 5S (*rrfA*)-23S (*rrlB*) rDNA intergenic spacer [50]. The PCR products were electrophoresed on a 1% agarose gel, stained with Gold View Nucleic Acid Stain, and visualized with a UV transilluminator.

### RFLP analysis

The positive PCR products of the 5S-23S rDNA intergenic spacer regions were further analyzed by RFLP. Previously extracted DNA of *B. afzelii*, *B. garinii*, *B. valaisiana*, *B. burgdorferi *s.s. and *B. lusitaniae *were used as positive controls. For each positive sample 13 μl of amplified DNA were digested at 65°C overnight in a solution containing 5 U of Tru1 I (300 u/ml) and 1 × Buffer R (Fermentas). Electrophoresis was carried out in 16% polyacrylamide gel at 150 V for 3 h. The gels were stained with SYBR Gold nucleic acid gel stain (Molecular Probes, Leiden, The Netherlands) for 20 min, and bands were visualized with a UV transilluminator.

All procedures, DNA isolation, PCR, and electrophoresis were performed in separate rooms using different pipettes and racks, with separate lab coats and disposable gloves worn in each laboratory to prevent carry-over contamination and to avoid false-positive results. PCR mixture was prepared in a sterile PCR box. All liquid handling procedures were performed using disposable sterile filter tips. In each DNA isolation and PCR reaction, a negative control (water) was included.

### DNA sequencing of PCR products

Three randomly selected PCR products of 16S rDNA of the *Anaplasmataceae *family were sequenced in the Laboratory of Biomedical Microbiology and Immunology at the University of Veterinary Medicine and Pharmacy in Košice. Sequencing only a few selected samples to exactly verify a bacterium species is an acceptable procedure [14]. Prior to the sequencing, PCR products were purified using a QIAquick PCR purification kit (Qiagen). The complementary strands of each sequenced product were manually assembled.

### Index of co-infection (I_c_)

Ginsberg [38] developed an index of co-infection (I_c_), which quantify the degree of departure of the number of mixed infections from independence. This is defined as the difference of the number of co-infections from the number expected due to chance alone, as a percentage of the total number of infected ticks in the sample.

$${\text{I}}_{\text{c}}=\left[\left(\text{O}-\text{E}\right)∕\text{N}\right]\times 100,$$

where: O = number of observed coinfections, E = expected number of co-infected ticks due to chance alone, N = total number of ticks infected by either or both microorganisms.

E=[(a+b)(a+c)]∕(a+b+c+d),

N=a+b+c,

where: a = number of ticks infected with both bacteria (equals O), b = number of ticks infected only with microorganism 1, c = number of ticks infected only with microorganism 2, and d = number of ticks not infected with either microorganism. Ic is positive when the number of co-infections is greater than expected, and negative when there are fewer co-infections than would be expected due to chance alone. Significance of the index was calculated by a chi-square test.

### Statistical analysis

To improve sample size and show more general patterns, data from the two breeding seasons (2008 and 2009) were pooled. Statistics were performed using SPSS for Windows, and all tests are two-tailed. Confidence limits (95% CL) for binary, presence-absence, data were calculated in an Excel macro.

## Competing interests

The authors declare that they have no competing interests.

## Authors' contributions

AE collected data, performed field and laboratory work, analysed data and wrote initial draft. KD and ZS collected data and performed field and laboratory work. PT analysed data and wrote initial draft. VM and IM supervised the laboratory work and intellectually support the study. All authors read and approved the final manuscript.
